# Supplementary material for: Lipopolysaccharide is Inserted into the Outer Membrane through An Intramembrane Hole, A Lumen Gate, and the Lateral Opening of LptD
Source: Structure. 2015 Mar 3;23(3):496–504. doi: 10.1016/j.str.2015.01.001 (PMC4353691; doi:10.1016/j.str.2015.01.001)
Supplement: Document S1. Figures S1–S4 [file mmc1.pdf]

Structure, Volume *23*

Supplemental Information

Lipopolysaccharide is Inserted into the Outer Membrane through An Intramembrane Hole, A Lumen Gate, and the Lateral Opening of LptD

Yinghong Gu, Phillip J. Stansfeld, Yi Zeng, Haohao Dong, Wenjian Wang, and Changjiang Dong

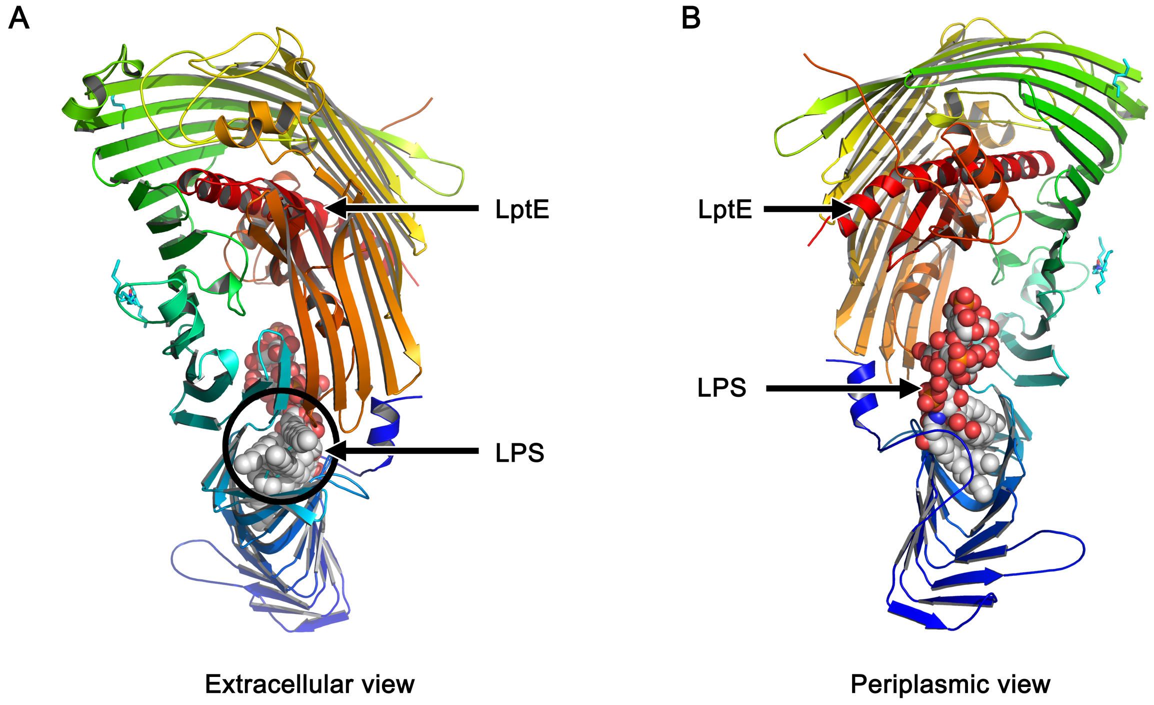

Figure S1, related to Figure 1. LPS molecule is modeled into the LptD/E complex. The modeling is based on the positions of detergent molecules LDAO and C8E4 in the N-terminal domain of LptD. (*A*) The extracellular view of the LptD/E complex with LPS. The acyl tails of the LPS are pointing to the intramembrane hole, which is shown in a dark circle. (*B*) The periplasmic view of the LptD/E complex with LPS. The hydrophobic portion of LPS is located in the hydrophobic core of the N-terminal domain, while the rest of portions of LPS are stretched out for entering the barrel. The LptE may assist core oligosaccharide and O-antigen transport within the barrel.

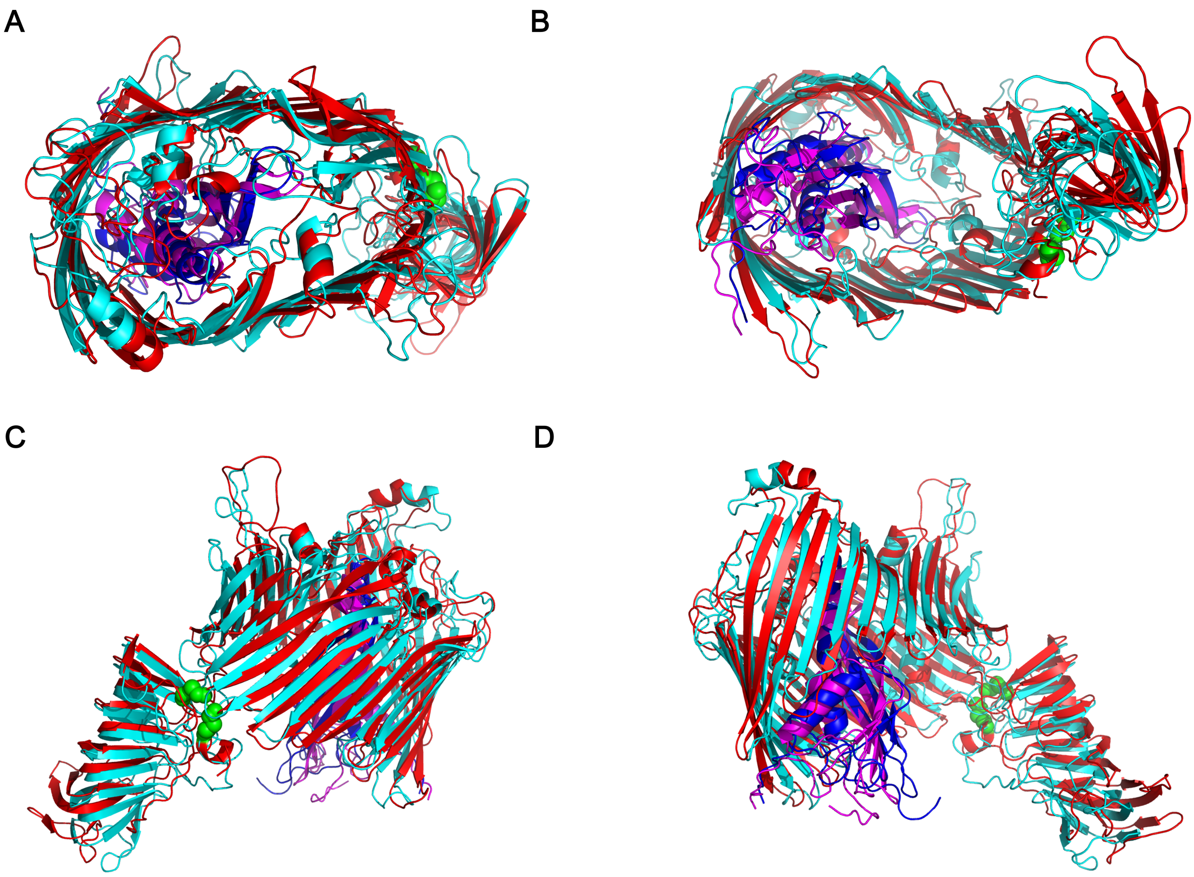

Figure S2, related to Figure 2. Comparison of conformational changes of the oxidized and reduced LptD/E after 100s molecular dynamics simulations at 350K. The oxidized LptD/E are in red and magenta, while the reduced LptD/E are in cyan and blue, respectively. The two disulfide bonds are shown in green. (A) The extracellular view of the LptD/E complex. Strands β25, β26 and their extracellular loop of the reduced LptD/E are sealed the pore, which is shown in a black dotted circle. (B) The periplasmic view of the LptD/E complex. The different conformational changes between the reduced and oxidized LptD/E at the N-terminal strands, shown in a black dotted circle. (C). A side view of the LptD/E complex. The conformational changes between the reduced and oxidized forms of LptD/E around the disulfide bonds, shown in a black dotted circle. The N-terminal helix in the reduced form of LptD/E become a loop. (D) Another side view of the LptD/E complex. The LptE in the oxidized form moves toward one side of the lumen, which makes the barrel widely open for LPS translocation, shown in a black dotted line. The Fig. C rotates 180 degree along y-axis to become Fig. D.

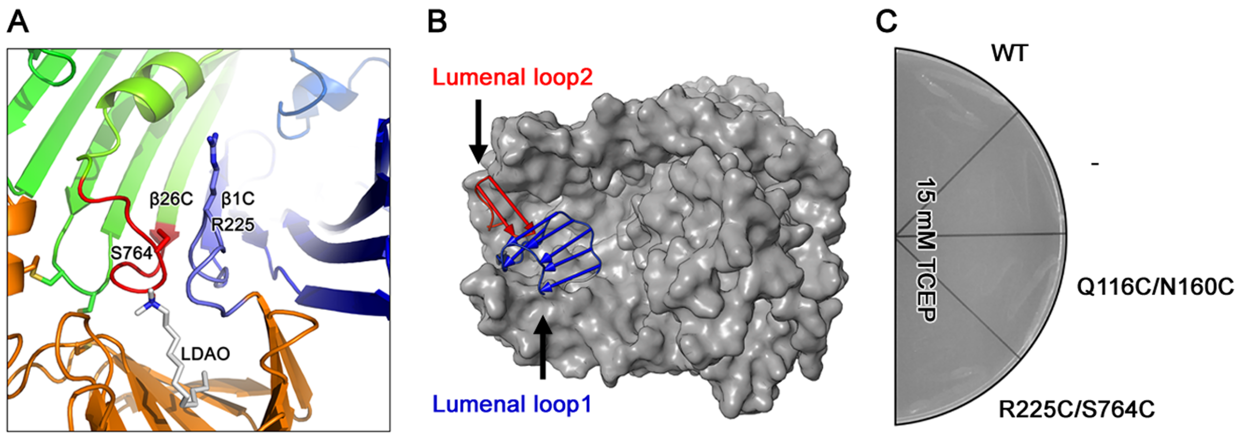

Figure S3, related to Figure 3. The lumen gate of LptD, and The *E. coli* cells with the LptD wild type and cysteine mutants Q116C/N160C and R225C/S764C are killed or inhibited by 15 mM TCEP. (*A*) The lumen gate are two loops in blue and red. There is a LDAO molecule, LPS's mimetics, in front of the lumen gate. Residues S764 and R225 are shown in stick. (*B*) The switch of the lumen gate. The arrows show the lumen gate from an open position to a closed position, which is based on the LptD/E structures of *S. typhimurium* LT2 and *S. flexneri*. (C) The TCEP at 15 mM may break the disulfide bond between C173 and C727, which is in the membrane and essential for the vitality of *E. coli*.

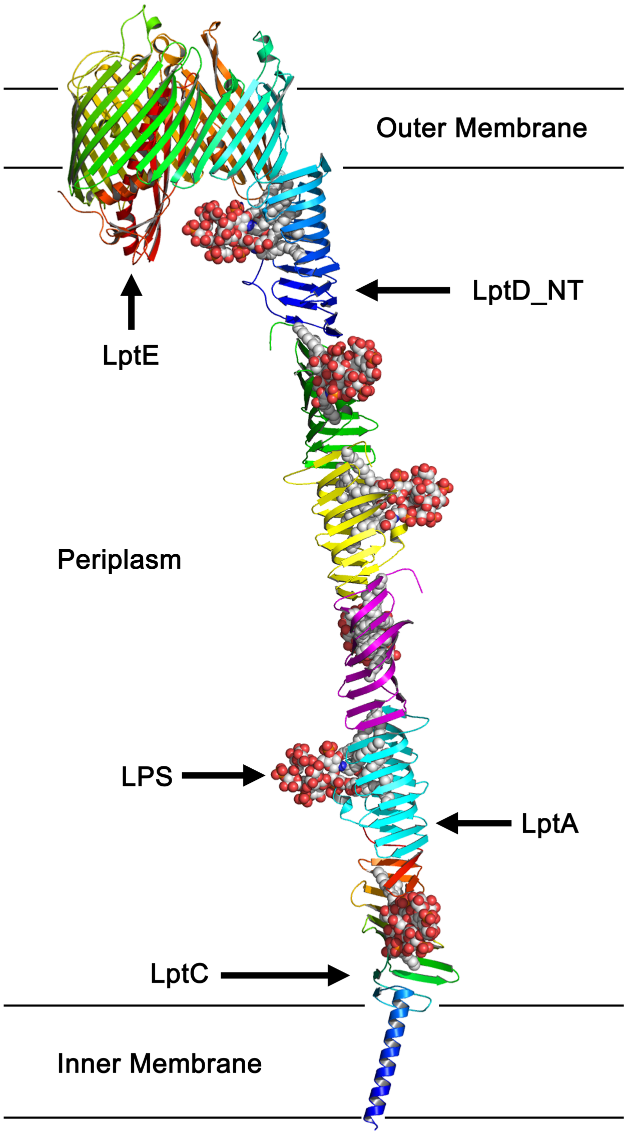

Figure S4, related to Figure 4. **The LPS transport slide formed by LptC, LptA and LptDE across the periplasm**. The slide contains one LptC, four LptA and one LptD molecule(s). The slide rotates ~360° from the LptC to LptD. The LPS molecules are modeled in the slide to show the rotation of the slide.

Video S1, related to Figure 2. The molecular dynamics simulation of oxidized LptD/E complex. The β1C in red and β26C in green. The hydrophobic residues in the intramembrane hole are in blue.
