# Supplementary figures and images for: Lipopolysaccharide is Inserted into the Outer Membrane through An Intramembrane Hole, A Lumen Gate, and the Lateral Opening of LptD
Source: Structure. 2015 Mar 3;23(3):496–504. doi: 10.1016/j.str.2015.01.001 (PMC4353691; doi:10.1016/j.str.2015.01.001)

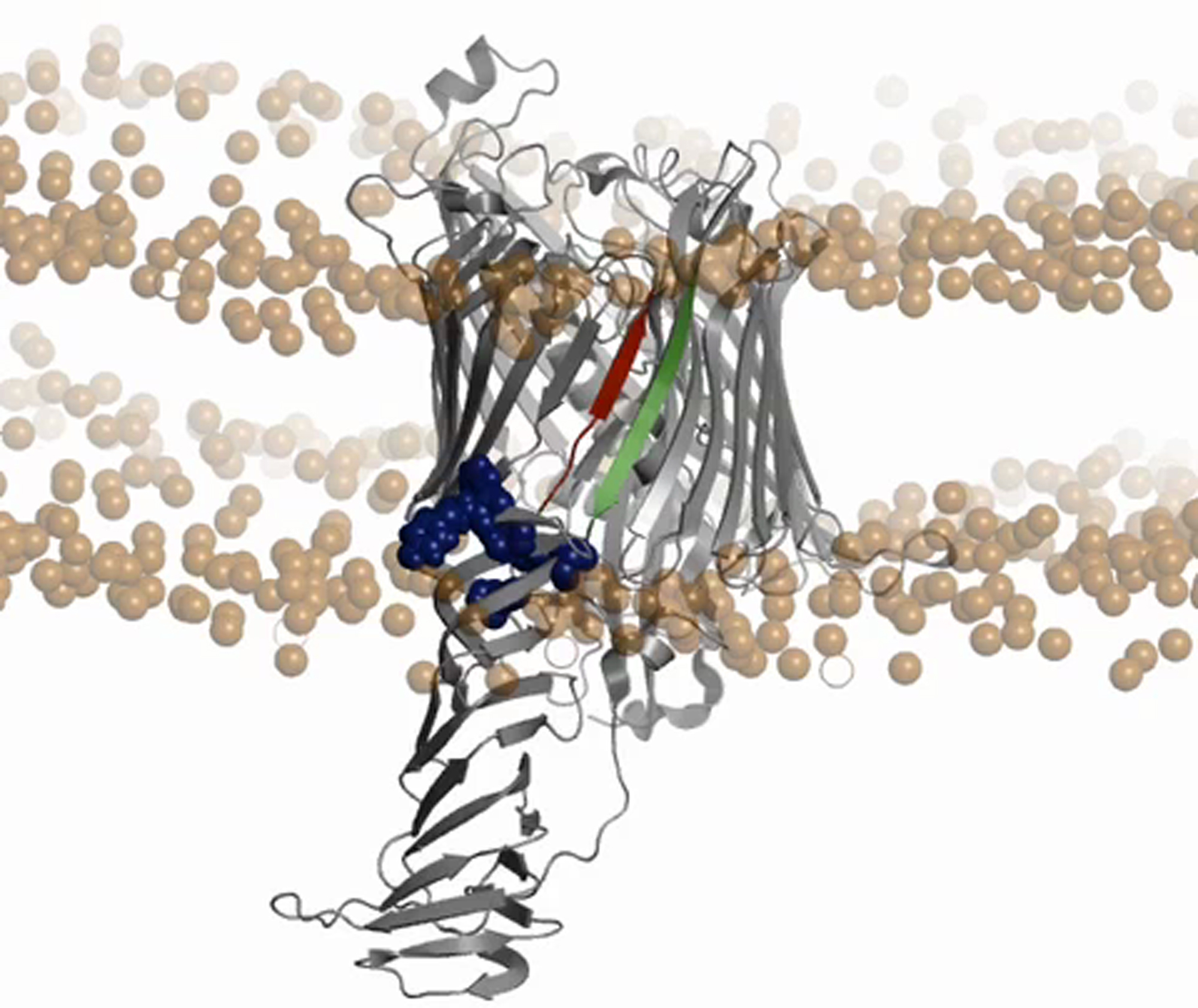

Supplement: Video S1. The MD Simulation of Oxidized LptD/E ComplexThe β1C in red and β26C in green; the hydrophobic residues in the intramembrane hole are in blue [file mmc4.jpg]
