# Supplementary material for: Lipopolysaccharide is Inserted into the Outer Membrane through An Intramembrane Hole, A Lumen Gate, and the Lateral Opening of LptD
Source: Structure. 2015 Mar 3;23(3):496–504. doi: 10.1016/j.str.2015.01.001 (PMC4353691; doi:10.1016/j.str.2015.01.001)
Supplement: Document S2. Article plus Supplemental Information [file mmc5.pdf]

Article

Structure

Lipopolysaccharide is Inserted into the Outer Membrane through An Intramembrane Hole, A Lumen Gate, and the Lateral Opening of LptD

Graphical Abstract

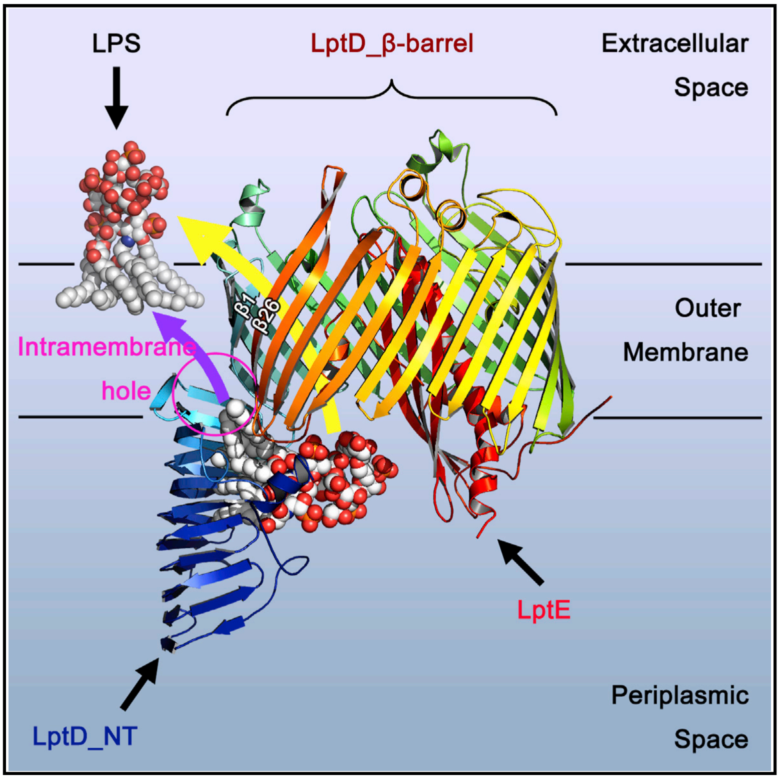

Highlights

- Hydrophobic residues at the N-terminal domain are essential for LPS transport
- A hydrophobic intramembrane hole of LptD is critical for LPS insertion
- A lumenal gate of LptD is important for translocation of LPS

Authors

Yinghong Gu, Phillip J. Stansfeld, ..., Wenjian Wang, Changjiang Dong

Correspondence

c.dong@uea.ac.uk (C.D.)
wenjian166@gmail.com (W.W.)

In Brief

Through molecular dynamics simulations, mutagenesis, and functional assays, Gu et al. reveal key residues of the N-terminal domain, a hydrophobic intramembrane hole, and a luminal gate of LptD for LPS transport, insertion, and translocation. These findings are significant not just for understanding the function of LptD, but also to develop novel antibiotics.

Gu et al., 2015, Structure *23*, 496–504
March 3, 2015 ©2015 The Authors
http://dx.doi.org/10.1016/j.str.2015.01.001

Structure
Article

Lipopolysaccharide is Inserted into the Outer Membrane through An Intramembrane Hole, A Lumen Gate, and the Lateral Opening of LptD

Yinghong Gu,[1] Phillip J. Stansfeld,[2] Yi Zeng,[1] Haohao Dong,[3] Wenjian Wang,[4,*] and Changjiang Dong[1,*]
[1]Biomedical Research Centre, Norwich Medical School, University of East Anglia, Norwich Research Park, Norwich NR4 7TJ, UK
[2]Department of Biochemistry, University of Oxford, South Parks Road, Oxford OX1 3QU, UK
[3]Biomedical Sciences Research Complex, School of Chemistry, University of St Andrews, North Haugh, St Andrews KY16 9ST, UK
[4]Laboratory of Department of Surgery, The First Affiliated Hospital, Sun Yat-sen University, 58 Zhongshan Road II, Guangzhou, Guangdong 510080, China
*Correspondence: c.dong@uea.ac.uk (C.D.), wenjian166@gmail.com (W.W.)
http://dx.doi.org/10.1016/j.str.2015.01.001
This is an open access article under the CC BY license (http://creativecommons.org/licenses/by/4.0/).

SUMMARY

Lipopolysaccharide (LPS) is essential for the vitality of most Gram-negative bacteria and plays an important role in bacterial multidrug resistance. The LptD/E translocon inserts LPS into the outer leaflet, the mechanism of which is poorly understood. Here, we report mutagenesis, functional assays, and molecular dynamics simulations of the LptD/E complex, which suggest two distinct pathways for the insertion of LPS. The N-terminal domain of LptD comprises a hydrophobic slide that injects the acyl tails of LPS directly into the outer membrane through an intramembrane hole, while the core oligosaccharide and O-antigen pass a lumen gate that triggers the unzipping of the lateral opening between strands β1C and β26C of the barrel of LptD, to finalize LPS insertion. Mutation of the LPS transport related residues or block of the LPS transport pathways results in the deaths of *Escherichia coli*. These findings are important for the development of novel antibiotics.

INTRODUCTION

All Gram-negative bacteria have an asymmetric outer membrane, in which the inner leaflet and the outer leaflet are formed by phospholipid and lipopolysaccharide (LPS), respectively (Whitfield and Trent, 2014; Ruiz et al., 2009). LPS contains three moieties: lipid A, core oligosaccharide, and O-antigen, forming a large amphipathic polymer. LPS is essential for the vitality of most Gram-negative bacteria and plays crucial roles not only in protecting the organisms from harsh environments and forming a biofilm but also in colonizing the human body and evading attacks from the human immune system (Whitfield and Trent, 2014; Zhang et al., 2013). Drug-resistant Gram-negative bacteria are becoming a global health threat and the outer membrane LPS plays an essential role in drug resistance. LPS forms a permeation barrier, which prevents hydrophobic antibiotics from entering the organisms, rendering antibiotics that are powerful against Gram-positive bacteria ineffective to Gram-negative bacteria (Ruiz et al., 2009; Zhang et al., 2013). The LPS transport proteins are attractive drug targets, as the impairment of LPS transport kills most of the Gram-negative bacteria (Srinivas et al., 2010; Sherman et al., 2013).

Lipid A with the core oligosaccharide and O-antigen units of LPS are synthesized in the cytoplasm and transported across the inner membrane to the periplasmic side of the inner membrane by MsbA and WzX, respectively, where the O-antigen is polymerized by WzY before being ligated to the core oligosaccharide on lipid A by WaaL to form a mature LPS molecule (Whitfield and Trent, 2014; Ruiz et al., 2009; Zhang et al., 2013). Seven LPS transport proteins, namely LptA, B, C, D, E, F, and G, form a transenvelope complex for LPS transport from the inner membrane to the outer leaflet of the outer membrane, of which the LptBFG proteins form an ABC transporter that extracts LPS from the inner membrane, passing it to another inner membrane protein called LptC, which then delivers it to the chaperone protein LptA (Sherman et al., 2013; Villa et al., 2013; Xiang et al., 2014; Freinkman et al., 2012; Tran et al., 2008). These processes require energy from ATP hydrolysis, which is achieved by LptB (Okuda et al., 2012; Sherman et al., 2014; Wang et al., 2014). The LptC, LptA, and N-terminal domain of LptD form a hydrophobic slide that ferries LPS across the periplasm. The LPS is then inserted into the outer leaflet of the outer membrane by the LPS translocon LptD/E complex (Chimalakonda et al., 2011; Chng et al., 2010; Freinkman et al., 2011; Grabowicz et al., 2013).

Two crystal structures of LptD/E complex from *Salmonella typhimurium LT2* and *Shigella flexneri* were reported, and both structures revealed that the LptD/E complex forms a novel two-protein barrel and plug architecture, with the LptDs forming a 26-stranded β-barrel that surrounds the LptE plug (Dong et al., 2014; Qiao et al., 2014; Bishop, 2014a). The N-terminal domain structure of LptD is similar to those of LptA and LptC (Qiao et al., 2014; Suits et al., 2008; Tran et al., 2010), which suggests that the N-terminal domain of LptD may be part of the slide for LPS transport from the inner membrane to the outer membrane (for clarity and consistency with the previous publications, we name the β strands of the LptD barrel as β1–26C and the β strands of the LptD N-terminal domain as β1–11a/bN, and the extracellular loops as L1–13 and periplasmic turns as T1–12). Our functional assays and molecular dynamics (MD) simulations suggest

that LptD inserts LPS into the outer membrane through a lateral opening between strands β1C and β26C (Dong et al., 2014). However, the precise LPS insertion mechanism by the LptD/E translocon is still not very clear. We performed further MD simulations, mutagenesis, and functional assays in this study, which revealed that LPS is inserted into the outer membrane through an intramembrane hydrophobic hole, a lumen gate with a novel switch, and the lateral opening between the strands β1C and β26C. Particularly, we identified the residues that are critical for LPS transport in the N-terminal domain of LptD and suggest that these residues interact with lipid A of LPS insertion during LPS insertion.

RESULTS

Modeling the N-Terminal Domain of LptD of *S. typhimurium LT2*

The crystal structures of LptD β-barrels from *S. typhimurium* and *S. flexneri* are very similar with root-mean-square deviation of 0.8225 over 523 Cα atoms. The sequence identity of the two proteins is 86.61%, which provides an excellent opportunity to model the LptD N-terminal of *S. typhimurium*. The model of LptD of *S. typhimurium* contains residues A25–M786 and the detergent molecules LDAO and C8E4, as observed in the *S. flexneri* LptD/E structure (Qiao et al., 2014).

N-Terminal Domain of LptD Transports LPS via An Inner Hydrophobic Slide

The N-terminal domain of LptD has a jellyroll-like structure, which resembles those of LptC and LptA. LptC, LptA, and the N-terminal domain of LptD form a consecutive transport slide, with a head to tail oligomerization, to transport LPS from the inner membrane to the outer membrane (Suits et al., 2008; Villa et al., 2013; Okuda et al., 2012; Dong et al., 2014). By incorporating an unnatural amino acid into the proteins and UV-dependent crosslinking, LPS and protein intermediates have been obtained at positions T47, F78, A172, and Y182 of LptC, and at positions T32, I36, F95, Y114, and L116 of LptA, suggesting that both LptC and LptA transport LPS through the hydrophobic core between the two β sheets (Okuda et al., 2012). The residues Y112, Y140, F170, and H189 in the core of the N-terminal domain of LptD bind detergent molecules, C8E4 and LDAO, which are potential mimetics of lipid A (Figures 1A and 1B), thus indicating that the LptD N-terminal domain transports LPS through its hydrophobic core by binding lipid A component of LPS in the same way as LptA and LptC. To confirm this, we generated single aromatic amino acid variants of the N-terminal domain of LptD and performed functional assays. The detergent binding residue variants Y140D, F170N, and F170G were lethal, and the H189G and Y112D variants impaired cell growth, while the single aromatic amino acid mutants Y63D in a loop, rather than in the hydrophobic core and F69N in the deep core, did not interfere with *Escherichia coli* growth (Figure 1C; Table S1). The protein expression levels of the LptD variants and the wild-type in the cell membrane were similar, which strongly suggests that the residues that interact with the detergents are involved in the LPS transport (Figure 1D). Residues Q116 and N160 are located at two opposite loops across the hydrophobic core, and it was theorized that a variant with a double cysteine mutation Q116C/N160C is able to form a disulfide bond in the oxidative periplasm. This would lock the LPS transport slide and block LPS transport in the N-terminal domain (Figure 1B). Indeed, the double cysteine mutant was proven lethal, while the single mutants Q116C and N160C could grow similarly to the wild-type, as proven by similar protein expression levels in the respective cell membranes (Figures 1C and 1D). We also identified two positively charged residues, R145 and R191, which are present in the two adjacent loops either side of the hydrophobic slide (Figure 1B). We speculate that these two residues may be important for binding the negatively charged LPS. A single glutamic acid substitution of either R145E or R191E does not impair the *E. coli* growth; however, the double glutamic acid substitution R145E/R191E causes *E. coli* cell death, indicating that at least one of the highly positive charged arginine residues is required for LPS transport (Figure 1C). In summary, the N-terminal domain of LptD uses the conserved hydrophobic core to bind lipid A and transport LPS; blockage of the LPS transport slide or mutation of the transport residues will result in *E. coli* cell death.

Lipid A Is Inserted Directly into the Outer Membrane through An Intramembrane Hydrophobic Hole

The lumen of the LptD barrel is highly hydrophilic and therefore no hydrophobic path is apparent for the transport of the lipid A moiety of LPS within the water-filled barrel (Dong et al., 2014). MD simulations reveal the extent to which the N-terminal domain of LptD is inserted within a lipid membrane. This agrees with the orientation predicted by the orientations of proteins in the membrane PPM server (Figure 2A). The domain appears to act like a needle, perfectly positioned to inject the acyl tails directly into the hydrophobic plane of the bilayer and thereby shielding it from the polar head groups. Indeed, examination of the outer surface of the N-terminal domain of LptD revealed hydrophobic residues from F203 to Y215 at the outer interface of β11aN and β11bN, confirming that β11aN and β11bN are inserted in the hydrophobic bilayer of the outer membrane (Figure 2B). The intramembrane hole is highly hydrophobic, consisting of the N-terminal residues W180, F228, V208, F203, F211, L216, L218, and the C-terminal residues L760 and L763 (Figures 2C and 2D). The structure of acyl tails of lipid A is similar to the detergents LDAO and C8E4, and therefore, LPS can be modeled on the N-terminal domain by superimposing lipid A to the detergents, showing that lipid A is transported into the intramembrane hole from the narrow side of the LPS molecule (Figure S1). To confirm that the N-terminal domain is required for direct insertion into the outer membrane, we made a deletion removing the β11aN and β11bN (residues F203–Y215), and performed functional assays, which revealed that this deletion results in the deaths of the *E. coli* cells (Figure 2E). Tyrosine residues are known to be crucial for the anchoring of proteins in the membrane. We speculate that hydrophobic Y212 and Y215 are important for insertion of β11aN and β11bN in the outer membrane, and mutation of them to hydrophilic residues may destabilize the two β strands in the outer membrane. As expected, both variants Y215D and Y212D caused *E. coli* death (Figure 2E).

The Hydrophobic Residues that Comprise the Intramembrane Hole Are Essential

To test whether the hydrophobic residues found around the intramembrane hole are essential for the vitality of *E. coli* cells,

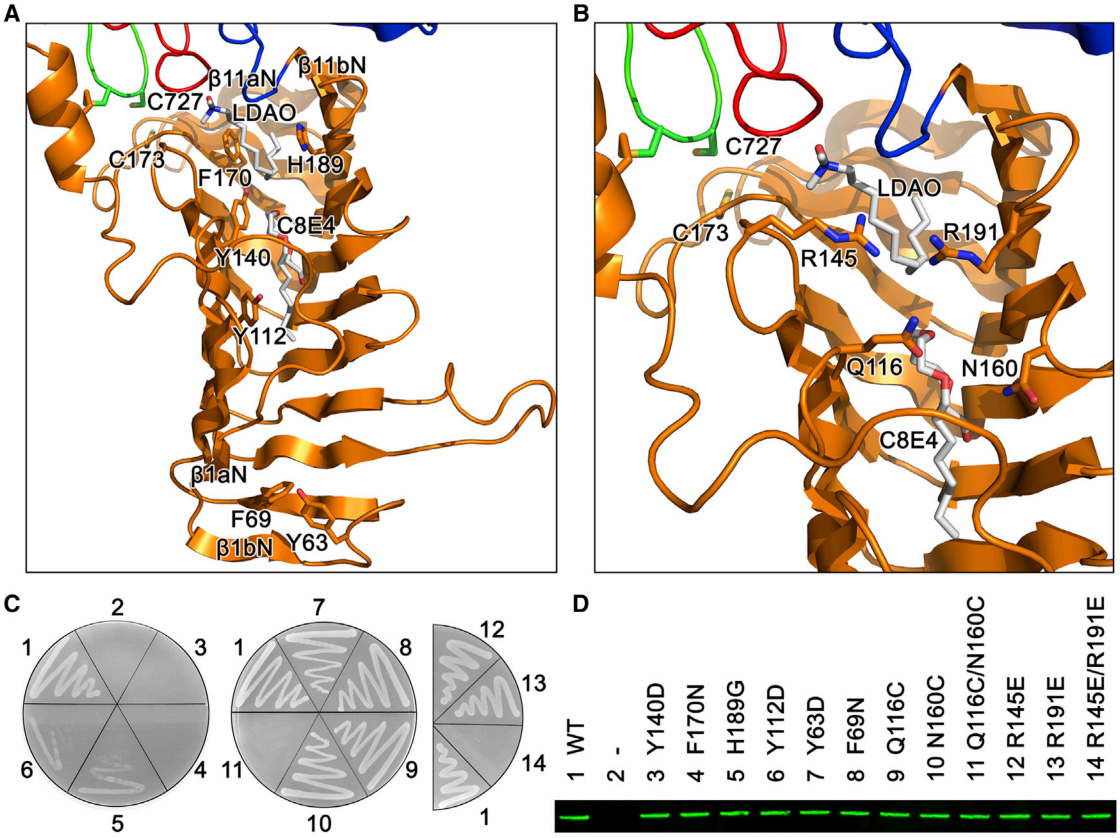

Figure 1. N-terminal Domain of LptD Forms a Hydrophobic LPS Transport Slide

The functional assays and the western blot shown here were repeated three times.

(A) Aromatic residues in the hydrophobic slide. Y112, Y140, F170, and H189 are involved in binding LDAO and C8E4, while Y63 and F69 do not appear to bind.

(B) Q116 and N160 are located in two loops above the LPS transport slide, and the double cysteine mutation is able to form a disulfide bond and block the LPS transport. R145 and R191 are located in two other loops and are likely to interact with the highly negatively charged LPS to position in the right orientation for transport.

(C) The functional assays of the LptD variants of the N-terminal domain. 1, 2, 3, 4, 5, and 6 are LptD depleted strain AM661 with pACYCDuet-1 containing wild-type LptD, empty pACYCDuet-1, LptD variants Y140D, F170N, H189G, and Y112D, respectively. 7, 8, 9, 10, 11 represent mutants Y63D, F69N, Q116C, N160C, and double mutant Q116C/N160C, respectively. 12, 13, 14 represent mutants R145E, R191E and double mutant R145E/R191E, respectively.

(D) Protein expression levels of the wild-type LptD and variants in the cell membrane were analyzed by western blot.

we performed the functional assays on a set of single amino acid variants. The absolutely conserved residue W180Q variant is lethal. In contrast, a highly conserved residue F228E variant does not show any defect in cell growth (Figure 2E; Table S1). Almost all other hydrophobic residue variants V208D, F203N, F211N, L218D, and L760D are lethal, and the L763D variant impairs cell growth (Figures 2E and 3C), despite the protein expression levels similar to that of variant F228E (Figures 2F and 3D). This is highly suggestive that the hydrophobic residues are critical for insertion of the lipid A of LPS into the outer membrane.

A Lumenal Gate Is Essential for Oligosaccharide and O-Antigen Translocation

The oligosaccharide and O-antigen are transported across the outer membrane through the barrel of LptD (Dong et al., 2014; Qiao et al., 2014). The in vitro and in vivo assays suggested that residues R91 and K136 of *E. coli* LptE may play a role in LPS transfer and disaggregation, while extracellular loop 4 of LptD interacts with LptE at this region (Malojcic et al., 2014), suggesting that the LptE may help to open the pore through the loop 4 to allow core oligosaccharide and O-antigen to emerge on the cell surface (Bishop, 2014b). To further explore this mechanism, we examined the lumen of the LptD barrel, where two loops at the bottom of the barrel were observed pointing towards its center. Lumenal loop 1 connects β1C of the LptD barrel to its N-terminal domain, consisting of residues V220 to I230, and lumenal loop 2 links β26C of the LptD barrel to its C terminus, comprising residues I758 to Y767 (Figures 3A and 3B). We propose that the two lumenal loops form a lumenal gate for oligosaccharide and O-antigen transport. Comparing LptD/E structures of *S. typhimurium* and *S. flexneri* revealed that the lumenal loop 1 is in a closed position in the LptD structure of *S. flexneri* and at an open position in that of *S. typhimurium*, while lumenal loop 2 is disordered in the LptD structure of *S. typhimurium* (Figures 3B and S3).

To check whether this gate is important, we made the deletions of lumenal loop 1 (residues V220–I230) or lumenal loop 2 (I758–Y767), with the result that both deletions led to *E. coli*

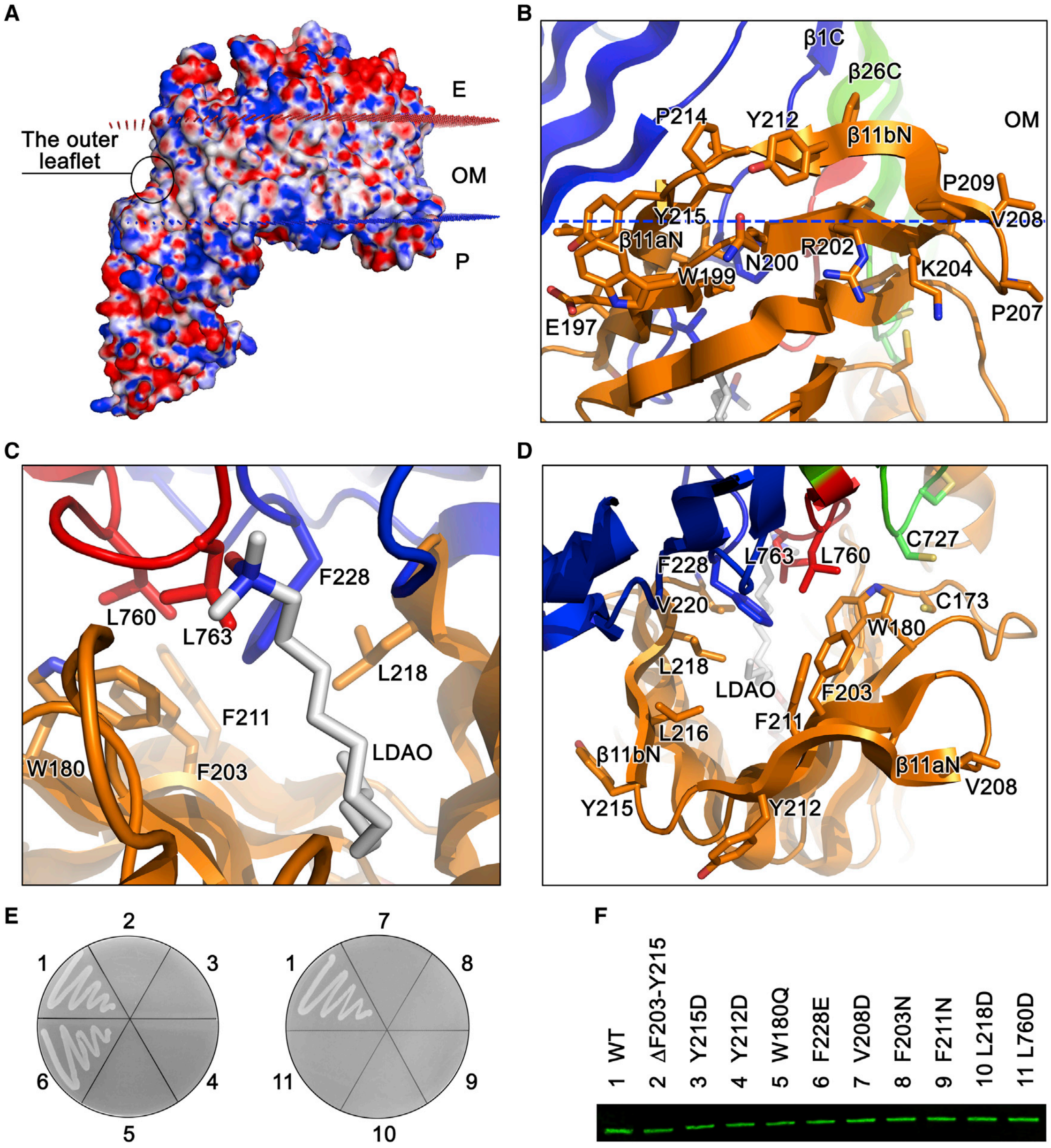

Figure 2. Intramembrane Hydrophobic Hole for LPS Insertion and the Essential Role of the Hydrophobic Residues for the Cell Vitality

The functional assays and the western blot shown here were repeated three times.

(A) Electrostatic potential map of LptD. Molecular simulations and membrane orientation analysis reveal that LptD forms an intramembrane hole. The location of the hole is marked by a black circle. E, OM, and P represent the extracellular side, the outer membrane, and the periplasmic side, respectively. The outer membrane core is between the red and the blue dotted lines.

(B) The N-terminal β11aN and β11bN are inserted into the outer membrane. The hydrophobic residues shown are found within the membrane, and the charged residues shown are in the periplasm. The periplasmic boundary of the outer membrane is shown as a blue dotted line.

(C) The hydrophobic hole from the lumen side of LptD. Hydrophobic residues W180, F203, F211, and L218 from the N-terminal domain and F228, L760, and L763 from the C-terminal domain form the hydrophobic hole.

(D) The hydrophobic hole from the membrane side (back).

(E) Functional assays. 1, 2, 3, 4, 5, 6, 7, 8, 9, 10, and 11 represent LptD wild-type, deletion F203-Y215, mutants Y215D, Y212D, W180Q, F228E V208D, F203N, F211N, L218D, and L760D, respectively.

(F) Western blot analysis of protein expression levels of the wild-type LptD and variants.

cell death (Figure 3C). There are two highly conserved residues, R224 and L229, within lumenal loop 1 (V220–I230), and we wondered whether they play an important role in LPS transport. The R224E, L229D, and L229G variants, however, did not impair cell growth, as well as other variants, V220S, K223E, R225E, S226V, F228E, and F228G (Figure 3C; Table S1). Single amino acid substitution of the residues in lumenal loop 2 showed that two variants, L760D and L763D, could be involved in formation

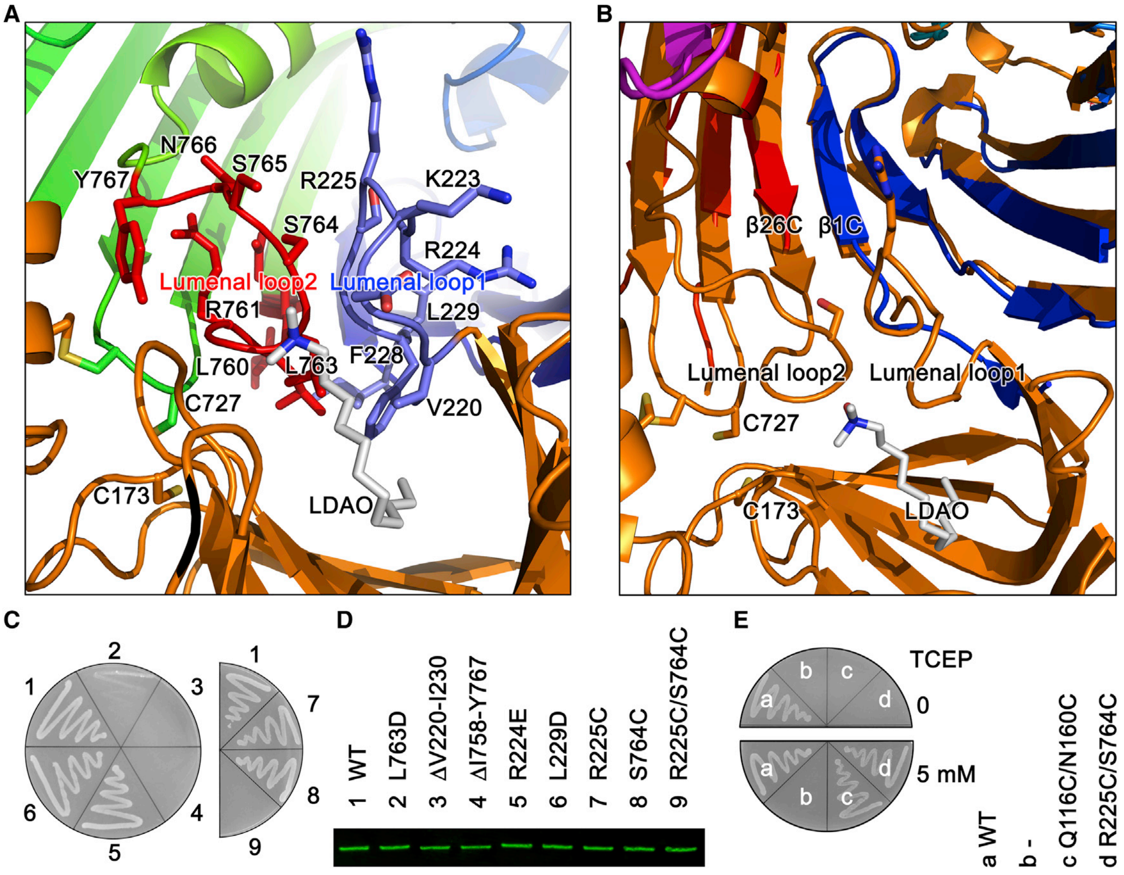

Figure 3. A Lumen Gate Is Important for Core Oligosaccharide and O-antigen Transport

The functional assays and the western blot shown here were repeated three times.

(A) Lumenal loop 1 and lumenal loop 2 in the lumen form a gate. The lumenal loop 1 and the residues are in blue, while lumenal loop 2 and the residues are in red.

(B) Superimposition of structures of *S. typhimurium* LptD and *S. flexneri* LptD reveals a novel switch for LPS transport in the barrel.

(C) Functional assays. 1, 2, 3, 4, 5, 6, 7, and 8 represent the LptD wild-type, L763D, deletion lumenal loop 1 (ΔV220-I230), deletion lumenal loop 2 (ΔI758-Y767), R224E, L229D, R225C, S764C, and double mutant R225C/S764C, respectively.

(D) Western blot analysis of the wild-type LptD and variants.

(E) The double cysteine substitutions can be rescued by TCEP at 5 mM. a, b, c, and d represent the LptD wild-type, empty vector pACYCDuet-1, double mutants Q116C/N160C, and R225C/S764C, respectively.

of the intramembrane hole and were lethal, while the other mutants, E759R, R761E, S764V, N766L, and Y767Q, did not impair cell growth on Luria broth (LB) agar plates (Table S1). Further C-terminal truncation experiments indicated that the residues in lumenal loop 2 may be important for the intramembrane hole formation: the C-terminal truncations ΔE757-M784, ΔE759-M784, ΔR761-M784, and ΔL763-M784 were lethal, while truncations ΔS765-M784, ΔY767-M784, ΔL769-M784, and ΔT771-M784 did not affect *E. coli* growth on LB agar plates (Table S1). To test whether the lumenal gate has to be open for LPS transport in the barrel, a double cysteine variant, R225C/S764C, was generated; the structure suggests that the mutant is able to form a disulfide bond and therefore prevents the lumenal gate opening (Figure S3). As expected, the R225C/S764C variant is lethal, while the single amino acid mutants R225C and S764C showed comparable growth with the wild-type LptD (Figure 3D). As the protein expression levels of the mutants in the membrane were found to be comparable with the wild-type, we conclude that the core oligosaccharide has to pass the lumenal gate inside the barrel for the LPS insertion. The hydrophobic residue variants Y247G, Y248G, and W249Q on strand β2C of the barrel did not cause defects in cell growth (Table S1), suggesting that they are not essential for LPS transport.

The two lumenal loops are in close proximity to a third periplasmic loop (T12) between β24C and β25C of the LptD barrel. This loop contains two highly conserved cysteine residues, C726 and C727, which couple to C173 and C31, respectively, and thereby stabilize the N-terminal domain of LptD in close proximity to the lumen switch and β1C and β26C lateral gate. Atomistic MD simulations of the oxidized and reduced forms of these disulphide bridges indicate that without the presence of the disulphide bridges, the N-terminal domain shows enhanced dynamics (Figure S2; Video S1). Specifically this includes increased mobility of the loop between β8bN and β9aN and the loss of the short helix at the very N-terminal portion of the structure. Furthermore, the loss of the disulphide bridges alters the conformation of β25C and β26C, which appears to induce

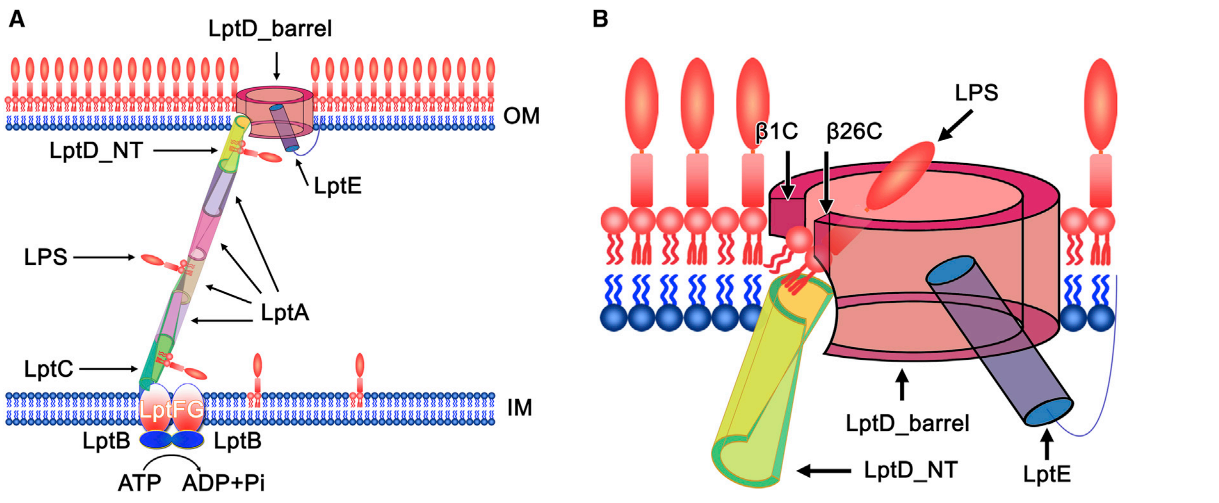

Figure 4. The Mechanism of LPS Insertion

(A) Seven LPS transport proteins form a transenvelope complex. One LptC molecule, four LptA molecules, and one N-terminal domain of LptD may form a ~360° rotation slide for LPS transport across the periplasm of *E. coli*. LPS is extracted from the inner membrane by LptBFG and passes it to LptC, while the LptC delivers LPS to LptA. LptC, LptA, and the N-terminal domain of LptD transport LPS through their hydrophobic cores. This process needs energy provided by LptB.

(B) Once in the N-terminal domain of LptD, lipid A of LPS is delivered into the outer membrane via the intramembrane hole, while the LptE may assist O-antigen to pass through the barrel of LptD. This process may also require energy. The insertion of lipid A into the outer membrane may trigger the lumen gate open for the core oligosaccharide into the barrel of LptD, promoting the lateral opening between the strands β1C and β26C to allow the core oligosaccharide to slide to the surface of the bacteria, where the divalent metal ions at the cell surface will bridge the highly negatively charged LPS molecules with the hydrophobic interactions among the lipid A portions of LPS to form the integrated outer membrane.

a closure of the outer mouth of the LptD barrel by the β25C-β26C loop (L13). In turn, this repositions the β26C to reinforce the closed state of the lateral gate of the barrel.

Reductant Tris(2-Carboxyethyl)phosphine Rescues the Double Cysteine Substitutions at 5 mM but Kills the Organisms at 15 mM

To further confirm that the double cysteine substitutions Q116C/N160C and R225C/S764C form disulfide bonds and block LPS transport, functional assays were performed in the presence of the reductant tris(2-carboxyethyl)phosphine (TCEP), which would break the disulfide bonds and rescue the mutants. LptD mutants Q116C/N160C or R225C/S764C could grow as well as the wild-type on LB agar plates containing 5 mM TCEP, which confirmed that TCEP could rescue the double cysteine substitutions Q116C/N160C and R225C/S764C at 5 mM (Figure 3E). However, either types of *E. coli*, with the mutants and the wild-type LptD, were killed or had their growth inhibited at 15 mM TCEP (Figure S3). We propose that the disulfide bond formed between Q116C and N160C in the periplasm, R225C and S764C in the lumen of LptD barrel are easier to be reduced than the disulfide bond between C173 and C727 in the core of the membrane. Disulfide bond formation between C173 and C727 is essential for the vitality of *E. coli* cells (Chng et al., 2012; Ruiz et al., 2010), and therefore, we speculate that TCEP may have broken this disulfide bond at the 15 mM concentration.

DISCUSSION

Thousands of LPS molecules are transported from the inner membrane to the outer leaflet of the outer membrane in a growing Gram-negative bacterial cell by the seven transenvelope LPS transport protein complex. In this complex, LptC, LptA, and the LptD N-terminal domain form the slide that transports LPS across the aqueous periplasm, with their hydrophobic cores interacting with lipid A of LPS. The width of the periplasm of *E. coli* is around 210 ± 27 Å (21 nm) (Matias et al., 2003), which suggests that the slide probably contains one LptC (~38 Å), four LptAs (~150 Å), and one LptD (~50 Å) (Qiao et al., 2014; Suits et al., 2008; Tran et al., 2010) molecule(s), arranged in a head to tail fashion (Figure S4). The slide is twisted and is comparable with rotation stairs or DNA helix. Each component of the slide rotates about 60°, and the whole slide rotates around 360°, which indicates that LPS rotates, perhaps to avoid aggregation, as it is transported across the periplasmic space (Figure 4A). The crystal structure of *S. flexneri* LptD showed that the detergent molecules, C8E4 and LDAO, were bound inside the hydrophobic core by Y112, Y140, F170, and H189, which strongly suggested that LPS was being transported along the hydrophobic slide via interactions with the acyl tails of lipid A moiety. The variants of the hydrophobic residues Y112D, Y140D, F170N, and H189G caused cell deaths or impaired cell growth, while aromatic residues Y63 and F69 may not be involved in binding LPS, as their mutants did not impair cell growth. These data suggest that LPS is transported along the LptD N-terminal hydrophobic core, which also indicates that LptA and LptC may use a similar strategy to transport LPS (Okuda et al., 2012). Q116 and N160 are located on two loops across the hydrophobic core, and the double variant Q116C/N160C is able to form a disulfide bond and block the LPS transport slide. The double mutant Q116C/N160C resulted in cell death, further corroborating that LPS is transported along the hydrophobic slide. Furthermore, TCEP could rescue the double mutant Q116C/N160C at 5 mM, which further proved the disulfide bond formation.

LPS is a macromolecule containing the large hydrophobic lipid A, and hydrophilic oligosaccharide and polysaccharide (O-antigen). It is a great challenge to use a water-filled barrel to transport hydrophobic molecules across the outer membrane. Several outer membrane proteins FadL, PagP, OmpW, and OprG adopt a lateral opening mechanism to diffuse the hydrophobic molecules to the outer membrane (van den Berg et al., 2004; Hearn et al., 2009, Hong et al., 2006; Khan and Bishop, 2009; Touw et al., 2010). How does LptD/E complex precisely and selectively insert LPS, an amphipathic molecule, into the outer leaflet of the outer membrane? Previous studies suggested that LptD uses a lateral opening or exit portal to insert the LPS into the outer membrane, with the core oligosaccharide and O-antigen being transported through the LptD barrel (Dong et al., 2014; Qiao et al., 2014). The β11aN and β11bN of the N-terminal domain are inserted into the outer membrane, forming the intramembrane hydrophobic hole with loops from β1C and β26C of the C-terminal barrel, which directly delivers lipid A of LPS into the outer membrane. The hydrophobic residues W180, F203, V208, F211, L218, L760, and L763, which are involved in the hydrophobic hole formation, are critical, and their substitutions caused the deaths of *E. coli* cells or impaired cell growth, indicating the importance of these residues in LPS transport and insertion. We speculate that the two lumenal loops form the lumenal gate for core oligosaccharide transport inside the barrel. R145 and R191 are close to the lumen gate. At least one arginine (either R145 or R191) is required to interact with the negatively charged LPS, keeping it at the correct orientation for entry into the barrel through the lumen gate. The core oligosaccharide and O-antigen may move through the LptD barrel using a similar mechanism to Wza or AlgE (Dong et al., 2006; Whitney et al., 2011) with the help of LptE. The double mutant S764C/R225C is able to form a disulfide bond and prevents the lumen gate from opening, which in turn results in *E. coli* death. This proposition was confirmed when TCEP rescued the LptD mutant at 5 mM.

We propose that lipid A is transported from the N-terminal slide of LptD to the intramembrane hole (Figure 4). At the same time, the core oligosaccharide and O-antigen moieties of LPS translocate within the LptD barrel, facilitated by LptE (Malojcic et al., 2014). We speculate that as lipid A reaches the intramembrane tip of the N-terminal domain, the core oligosaccharide slides past the lumenal gate switch, between β1C and the C-terminal end of β26C. This initiates the separation of the lateral gate between β1C and β26C. Once the lipid A tails enter the membrane and the remainder of the core oligosaccharide is translocated, the LPS molecule acts as a zip-slider, unzipping the H-bonds between β1C and β26C. After the β1C and β26C lateral gate is unlocked, the remainder of the LPS molecule can then slide from the center of the porin to join the lipid A molecule in the membrane with the help of LptE. Nevertheless, our data cannot exclude a different order of events, whereby lipid A is first inserted into the outer membrane through the intramembrane hole. This then promotes the core oligosaccharide to enter into the lumen gate and triggers the lateral opening between β1C and β26C. These processes insert the lipid A into the outer membrane and pull the O-antigen through the LptD barrel to the bacterial outer surface. Further investigations are required to confirm whether these processes of LPS translocation within LptD and LPS insertion require energy and what the role the LptE plays (Malojcic et al., 2014; Bos and Tommassen, 2011). It is proposed that once the highly negatively charged LPS (core oligosaccharide) reaches the positively charged surface, which is rich in divalent metal ions, these ions would bridge the LPS molecules together to form an integral hydrophilic membrane barrier (Dong et al., 2014; Bishop, 2014a). In addition, the lumenal loop 1, loop 2, and disulfide bond formation between C173 and C727 ensures that the LPS is correctly delivered into the gate between strands β1C and β26C of LptD rather than other strands, thereby preventing mislocation of LPS (Dong et al., 2014).

In summary, through MD simulations, mutagenesis, and functional assays, we have confirmed that the N-terminal domain of LptD transports LPS via the hydrophobic core. The lipid A moiety of LPS is directly inserted into the outer membrane by the intramembrane hydrophobic hole consisting of β11aN, β11bN, β1C, and β26C, while the O-antigen is delivered through the barrel of LptD by the assistance of LptE. The core oligosaccharide goes through the lumen gate comprising two lumenal loops from β1C and β26C, which may induce the lateral opening between β1C and β26C, thereby allowing the core oligosaccharide to reach the extracellular cell surface through extracellular loops (Bishop, 2014b). These findings are significant not just for understanding LPS insertion but also to facilitate the development of drugs that combat highly resistant Gram-negative bacteria.

EXPERIMENTAL PROCEDURES

Molecular Modeling

Modeller v9.9 (Sali and Blundell, 1993) was used to build molecular models of the reduced and oxidized states of the *Salmonella typhimurium* LptD/E structure, and to incorporate the N-terminal domain from the *Shigella flexneri* LptD/E structure.

MD Simulations

All MD simulations were performed using GROMACS v5.0 (Pronk et al., 2013). The Martini 2.2 force field was used to run the initial 1 μs coarse grained (CG) MD simulations to enable the assembly and equilibration of a dimyristoylphosphatidylglycerol:dimyristoylphosphatidy-lethanolamine bilayer around the LptD/E complexes (de Jong et al., 2013; Stansfeld et al., 2013). The 1 μs snapshots of the CG simulations were then converted to atomic detail with the atomistic protein structure aligned with the CG protein within the assembled lipid bilayer (Stansfeld and Sansom, 2011). The systems were then equilibrated further for 1 ns with the protein restrained, before 100 ns of unrestrained atomistic MD at 350 K using the Gromos53a6 force field (Oostenbrink et al., 2004). All systems were neutralized with a 150 mM concentration of NaCl.

Mutagenesis

In order to detect the protein expression using western blot, a construct harboring the *lptD* gene of *S. typhimurium* strain LT2 was generated as described previously (Dong et al., 2014). In brief, the *lptD* gene fragment was cloned into pACYCDuet-1 (Novagen) between NdeI and XhoI sites, and a hexa-His tag was introduced into LptD between amino acids 27 and 28. Site-directed mutagenesis was performed according to a previously described protocol (Liu and Naismith, 2008) with Q5 Hot Start High-Fidelity DNA Polymerase (New England Biolabs). The primers used to generate single, double, deletion, and truncation mutants are listed in Table S2. All variants were confirmed by sequencing.

Functional Assays

Salmonella lptD mutants were used for functional assays in the *E. coli lptD* depleted strain AM661 (Sperandeo et al., 2008) as described previously

(Dong et al., 2014). Plasmids with empty vector pACYCDuet-1, wild-type *lptD*, and its different variants were transformed into *E. coli* AM661, respectively, and then grown on LB agar plates supplemented with antibiotics (50 μg ml^{-1} kanamycin and 34 μg ml^{-1} chloramphenicol), and with or without *L*-arabinose (0.2%). Single-colony was inoculated into 5 ml of LB medium supplemented with antibiotics and *L*-arabinose (*L*-arabinose was added only if the cell cannot grow or grows slowly without it) and incubated overnight, then streaked onto LB agar plates supplemented with antibiotics and with or without *L*-arabinose for functional assay (Table S1). To investigate whether the reductant rescued the double cysteine substitutions, the LB agar plates were supplemented with antibiotics and TCEP (Sigma-Aldrich).

Immunoblot Analysis

Western blotting was performed as described previously (Dong et al., 2014). Overnight cultures were pelleted and resuspended in Tris-buffered saline (TBS, 20 mM Tris, 150 mM NaCl, [pH 8.0]) and the cells were lysed by sonication. The lysate was centrifuged at 7,000 × *g* for 15 min at 4°C to remove the cell debris. The resulting supernatant was ultracentrifuged at 100,000 × *g* for 1 hr at 4°C, and the pelleted membrane fraction was resuspended in TBS containing 1% (w/v) *N*-lauroylsarcosine sodium salt (Sigma-Aldrich) to solubilize the inner membrane for 1 hr at 4°C. The outer membrane fraction was pelleted by ultracentrifugation as described above and resuspended in TBS. The protein sample was mixed with SDS-PAGE loading buffer and heated at 90°C for 5 min. Equivalent amounts of protein from each sample were separated on NuPAGE Novex 4%–12% Bis-Tris Protein Gels (Life Technologies) and then transferred onto a polyvinylidene fluoride membrane (Millipore), which was then blocked overnight at 4°C in protein-free T20 blocking buffer (Fisher Scientific). After blocking, the membrane was incubated with His•Tag Monoclonal Antibody (1:1,000, Millipore) diluted in buffer containing half blocking buffer and half PBS with 0.1% Tween 20 (PBS-T) for 1 hr at room temperature followed by washing four times with PBS-T, and then incubated with diluted secondary antibody (IRDye 800CW goat anti-mouse IgG) (1:5,000, LI-COR) for 1 hr at room temperature. The membrane was washed in PBS-T four times and in PBS two times, respectively. Images were acquired using the LI-COR Odyssey Infrared Imaging System (LI-COR).

SUPPLEMENTAL INFORMATION

Supplemental Information includes two tables, four figures, and one video and can be found with this article online at http://dx.doi.org/10.1016/j.str.2015.01.001.

AUTHOR CONTRIBUTIONS

C.D. and W.W. designed the research; Y.G., Y.Z., H.D., and W.W. performed the experiments and data analysis; P.J.S. performed the MD simulations and analyzed the data. C.D., W.W., Y.G., and P.J.S. wrote the manuscript.

ACKNOWLEDGMENTS

C.D. is a recipient of the Wellcome Trust New Investigator Award. P.J.S. is supported by BBSRC grant BB/I019855/1 and W.W. is supported by China Natural Science Foundation of Guangdong Province grant (S2013010016539). We thank Prof. Thomas J. Silhavy for providing the *E. coli* AM661 strain.

Received: October 29, 2014
Revised: December 29, 2014
Accepted: January 6, 2015
Published: February 12, 2015

REFERENCES

Bishop, R.E. (2014a). Structural biology: lipopolysaccharide rolls out the barrel. Nature *511*, 37–38.

Bishop, R.E. (2014b). Emerging roles for anionic non-bilayer phospholipids in fortifying the outer membrane permeability barrier. J. Bacteriol. *196*, 3209–3213.

Bos, M.P., and Tommassen, J. (2011). The LptD chaperone LptE is not directly involved in lipopolysaccharide transport in *Neisseria meningitidis*. J. Biol. Chem. *286*, 28688–28696.

Chimalakonda, G., Ruiz, N., Chng, S.S., Garner, R.A., Kahne, D., and Silhavy, T.J. (2011). Lipoprotein LptE is required for the assembly of LptD by the beta-barrel assembly machine in the outer membrane of *Escherichia coli*. Proc. Natl. Acad. Sci. USA *108*, 2492–2497.

Chng, S.S., Ruiz, N., Chimalakonda, G., Silhavy, T.J., and Kahne, D. (2010). Characterization of the two-protein complex in *Escherichia coli* responsible for lipopolysaccharide assembly at the outer membrane. Proc. Natl. Acad. Sci. USA *107*, 5363–5368.

Chng, S.S., Xue, M., Garner, R.A., Kadokura, H., Boyd, D., Beckwith, J., and Kahne, D. (2012). Disulfide rearrangement triggered by translocon assembly controls lipopolysaccharide export. Science *337*, 1665–1668.

de Jong, D.H., Singh, G., Bennett, W.F.D., Arnarez, C., Wassenaar, T.A., Schafer, L.V., Periole, X., Tieleman, D.P., and Marrink, S.J. (2013). Improved parameters for the martini coarse-grained protein force field. J. Chem. Theory Comput. *9*, 687–697.

Dong, C., Beis, K., Nesper, J., Brunkan-Lamontagne, A.L., Clarke, B.R., Whitfield, C., and Naismith, J.H. (2006). Wza the translocon for *E. coli* capsular polysaccharides defines a new class of membrane protein. Nature *444*, 226–229.

Dong, H., Xiang, Q., Gu, Y., Wang, Z., Paterson, N.G., Stansfeld, P.J., He, C., Zhang, Y., Wang, W., and Dong, C. (2014). Structural basis for outer membrane lipopolysaccharide insertion. Nature *511*, 52–56.

Freinkman, E., Chng, S.S., and Kahne, D. (2011). The complex that inserts lipopolysaccharide into the bacterial outer membrane forms a two-protein plug-and-barrel. Proc. Natl. Acad. Sci. USA *108*, 2486–2491.

Freinkman, E., Okuda, S., Ruiz, N., and Kahne, D. (2012). Regulated assembly of the transenvelope protein complex required for lipopolysaccharide export. Biochemistry *51*, 4800–4806.

Grabowicz, M., Yeh, J., and Silhavy, T.J. (2013). Dominant negative lptE mutation that supports a role for LptE as a plug in the LptD barrel. J. Bacteriol. *195*, 1327–1334.

Hearn, E.M., Patel, D.R., Lepore, B.W., Indic, M., and van den Berg, B. (2009). Transmembrane passage of hydrophobic compounds through a protein channel wall. Nature *458*, 367–370.

Hong, H., Patel, D.R., Tamm, L.K., and van den Berg, B. (2006). The outer membrane protein OmpW forms an eight-stranded beta-barrel with a hydrophobic channel. J. Biol. Chem. *281*, 7568–7577.

Khan, M.A., and Bishop, R.E. (2009). Molecular mechanism for lateral lipid diffusion between the outer membrane external leaflet and a beta-barrel hydrocarbon ruler. Biochemistry *48*, 9745–9756.

Liu, H., and Naismith, J.H. (2008). An efficient one-step site-directed deletion, insertion, single and multiple-site plasmid mutagenesis protocol. BMC Biotechnol. *8*, 91.

Malojcic, G., Andres, D., Grabowicz, M., George, A.H., Ruiz, N., Silhavy, T.J., and Kahne, D. (2014). LptE binds to and alters the physical state of LPS to catalyze its assembly at the cell surface. Proc. Natl. Acad. Sci. USA *111*, 9467–9472.

Matias, V.R., Al-Amoudi, A., Dubochet, J., and Beveridge, T.J. (2003). Cryo-transmission electron microscopy of frozen-hydrated sections of *Escherichia coli* and *Pseudomonas aeruginosa*. J. Bacteriol. *185*, 6112–6118.

Okuda, S., Freinkman, E., and Kahne, D. (2012). Cytoplasmic ATP hydrolysis powers transport of lipopolysaccharide across the periplasm in *E. coli*. Science *338*, 1214–1217.

Oostenbrink, C., Villa, A., Mark, A.E., and van Gunsteren, W.F. (2004). A biomolecular force field based on the free enthalpy of hydration and solvation: the GROMOS force-field parameter sets 53A5 and 53A6. J. Comput. Chem. *25*, 1656–1676.

Pronk, S., Pall, S., Schulz, R., Larsson, P., Bjelkmar, P., Apostolov, R., Shirts, M.R., Smith, J.C., Kasson, P.M., van der Spoel, D., et al. (2013). GROMACS 4.5: a high-throughput and highly parallel open source molecular simulation toolkit. Bioinformatics *29*, 845–854.

Qiao, S., Luo, Q., Zhao, Y., Zhang, X.C., and Huang, Y. (2014). Structural basis for lipopolysaccharide insertion in the bacterial outer membrane. Nature *511*, 108–111.

Ruiz, N., Kahne, D., and Silhavy, T.J. (2009). Transport of lipopolysaccharide across the cell envelope: the long road of discovery. Nat. Rev. Microbiol. *7*, 677–683.

Ruiz, N., Chng, S.S., Hiniker, A., Kahne, D., and Silhavy, T.J. (2010). Nonconsecutive disulfide bond formation in an essential integral outer membrane protein. Proc. Natl. Acad. Sci. USA *107*, 12245–12250.

Sali, A., and Blundell, T.L. (1993). Comparative protein modelling by satisfaction of spatial restraints. J. Mol. Biol. *234*, 779–815.

Sherman, D.J., Okuda, S., Denny, W.A., and Kahne, D. (2013). Validation of inhibitors of an ABC transporter required to transport lipopolysaccharide to the cell surface in *Escherichia coli*. Bioorg. Med. Chem. *21*, 4846–4851.

Sherman, D.J., Lazarus, M.B., Murphy, L., Liu, C., Walker, S., Ruiz, N., and Kahne, D. (2014). Decoupling catalytic activity from biological function of the ATPase that powers lipopolysaccharide transport. Proc. Natl. Acad. Sci. USA *111*, 4982–4987.

Sperandeo, P., Lau, F.K., Carpentieri, A., De Castro, C., Molinaro, A., Dehò, G., Silhavy, T.J., and Polissi, A. (2008). Functional analysis of the protein machinery required for transport of lipopolysaccharide to the outer membrane of *Escherichia coli*. J. Bacteriol. *190*, 4460–4469.

Srinivas, N., Jetter, P., Ueberbacher, B.J., Werneburg, M., Zerbe, K., Steinmann, J., Van der Meijden, B., Bernardini, F., Lederer, A., Dias, R.L., et al. (2010). Peptidomimetic antibiotics target outer-membrane biogenesis in *Pseudomonas aeruginosa*. Science *327*, 1010–1013.

Stansfeld, P.J., and Sansom, M.S.P. (2011). From coarse grained to atomistic: a serial multiscale approach to membrane protein simulations. J. Chem. Theory Comput. *7*, 1157–1166.

Stansfeld, P.J., Jefferys, E.E., and Sansom, M.S. (2013). Multiscale simulations reveal conserved patterns of lipid interactions with aquaporins. Structure *21*, 810–819.

Suits, M.D., Sperandeo, P., Deho, G., Polissi, A., and Jia, Z. (2008). Novel structure of the conserved gram-negative lipopolysaccharide transport protein A and mutagenesis analysis. J. Mol. Biol. *380*, 476–488.

Touw, D.S., Patel, D.R., and van den Berg, B. (2010). The crystal structure of OprG from *Pseudomonas aeruginosa*, a potential channel for transport of hydrophobic molecules across the outer membrane. PLoS One *5*, e15016.

Tran, A.X., Trent, M.S., and Whitfield, C. (2008). The LptA protein of *Escherichia coli* is a periplasmic lipid A-binding protein involved in the lipopolysaccharide export pathway. J. Biol. Chem. *283*, 20342–20349.

Tran, A.X., Dong, C., and Whitfield, C. (2010). Structure and functional analysis of LptC, a conserved membrane protein involved in the lipopolysaccharide export pathway in *Escherichia coli*. J. Biol. Chem. *285*, 33529–33539.

van den Berg, B., Black, P.N., Clemons, W.M., Jr., and Rapoport, T.A. (2004). Crystal structure of the long-chain fatty acid transporter FadL. Science *304*, 1506–1509.

Villa, R., Martorana, A.M., Okuda, S., Gourlay, L.J., Nardini, M., Sperandeo, P., Deho, G., Bolognesi, M., Kahne, D., and Polissi, A. (2013). The *Escherichia coli* Lpt transenvelope protein complex for lipopolysaccharide export is assembled via conserved structurally homologous domains. J. Bacteriol. *195*, 1100–1108.

Wang, Z., Xiang, Q., Zhu, X., Dong, H., He, C., Wang, H., Zhang, Y., Wang, W., and Dong, C. (2014). Structural and functional studies of conserved nucleotide-binding protein LptB in lipopolysaccharide transport. Biochem. Biophys. Res. Commun. *452*, 443–449.

Whitfield, C., and Trent, M.S. (2014). Biosynthesis and export of bacterial lipopolysaccharides. Annu. Rev. Biochem. *83*, 99–128.

Whitney, J.C., Hay, I.D., Li, C., Eckford, P.D., Robinson, H., Amaya, M.F., Wood, L.F., Ohman, D.E., Bear, C.E., Rehm, B.H., and Howell, P.L. (2011). Structural basis for alginate secretion across the bacterial outer membrane. Proc. Natl. Acad. Sci. USA *108*, 13083–13088.

Xiang, Q.J., Wang, H.Y., Wang, Z.S., Zhang, Y.Z., and Dong, C.J. (2014). Characterization of lipopolysaccharide transport protein complex. Cent. Eur. J. Biol. *9*, 131–138.

Zhang, G., Meredith, T.C., and Kahne, D. (2013). On the essentiality of lipopolysaccharide to Gram-negative bacteria. Curr. Opin. Microbiol. *16*, 779–785.

Structure, Volume *23*

Supplemental Information

Lipopolysaccharide is Inserted into the Outer Membrane through An Intramembrane Hole, A Lumen Gate, and the Lateral Opening of LptD

Yinghong Gu, Phillip J. Stansfeld, Yi Zeng, Haohao Dong, Wenjian Wang, and Changjiang Dong

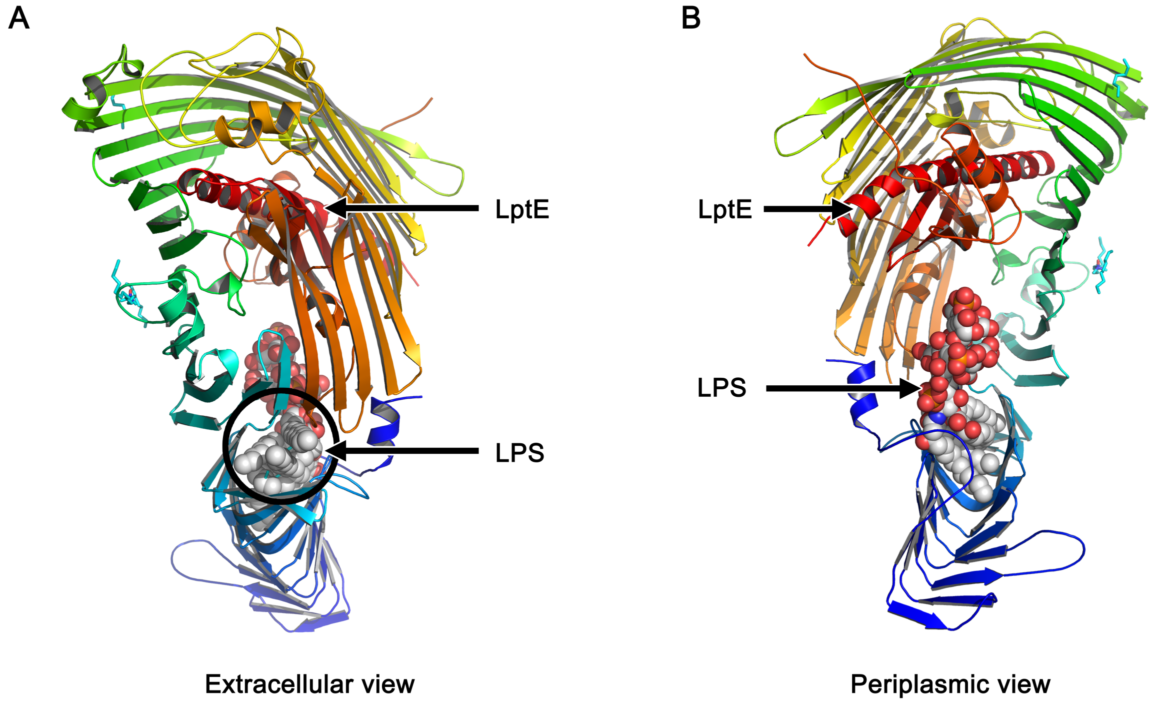

Figure S1, related to Figure 1. LPS molecule is modeled into the LptD/E complex. The modeling is based on the positions of detergent molecules LDAO and C8E4 in the N-terminal domain of LptD. (*A*) The extracellular view of the LptD/E complex with LPS. The acyl tails of the LPS are pointing to the intramembrane hole, which is shown in a dark circle. (*B*) The periplasmic view of the LptD/E complex with LPS. The hydrophobic portion of LPS is located in the hydrophobic core of the N-terminal domain, while the rest of portions of LPS are stretched out for entering the barrel. The LptE may assist core oligosaccharide and O-antigen transport within the barrel.

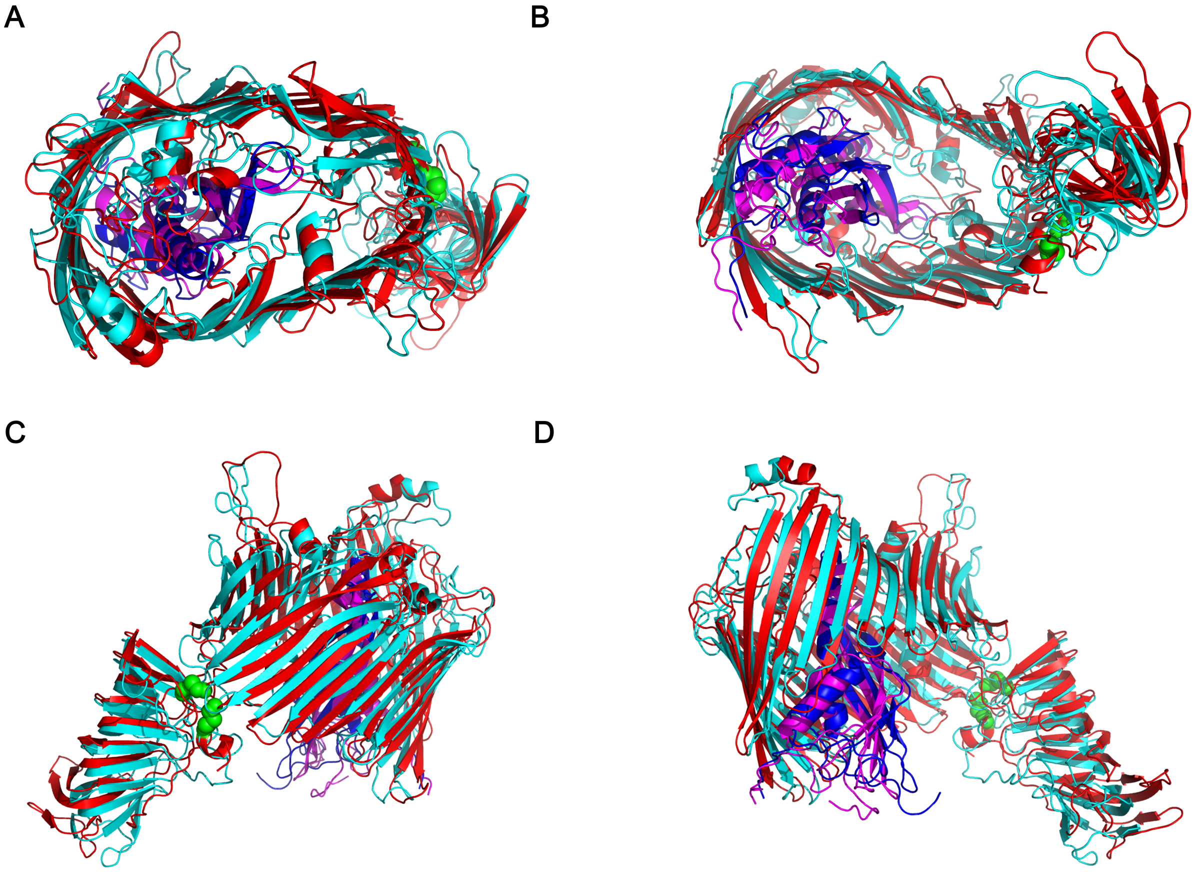

Figure S2, related to Figure 2. Comparison of conformational changes of the oxidized and reduced LptD/E after 100s molecular dynamics simulations at 350K. The oxidized LptD/E are in red and magenta, while the reduced LptD/E are in cyan and blue, respectively. The two disulfide bonds are shown in green. (A) The extracellular view of the LptD/E complex. Strands β25, β26 and their extracellular loop of the reduced LptD/E are sealed the pore, which is shown in a black dotted circle. (B) The periplasmic view of the LptD/E complex. The different conformational changes between the reduced and oxidized LptD/E at the N-terminal strands, shown in a black dotted circle. (C). A side view of the LptD/E complex. The conformational changes between the reduced and oxidized forms of LptD/E around the disulfide bonds, shown in a black dotted circle. The N-terminal helix in the reduced form of LptD/E become a loop. (D) Another side view of the LptD/E complex. The LptE in the oxidized form moves toward one side of the lumen, which makes the barrel widely open for LPS translocation, shown in a black dotted line. The Fig. C rotates 180 degree along y-axis to become Fig. D.

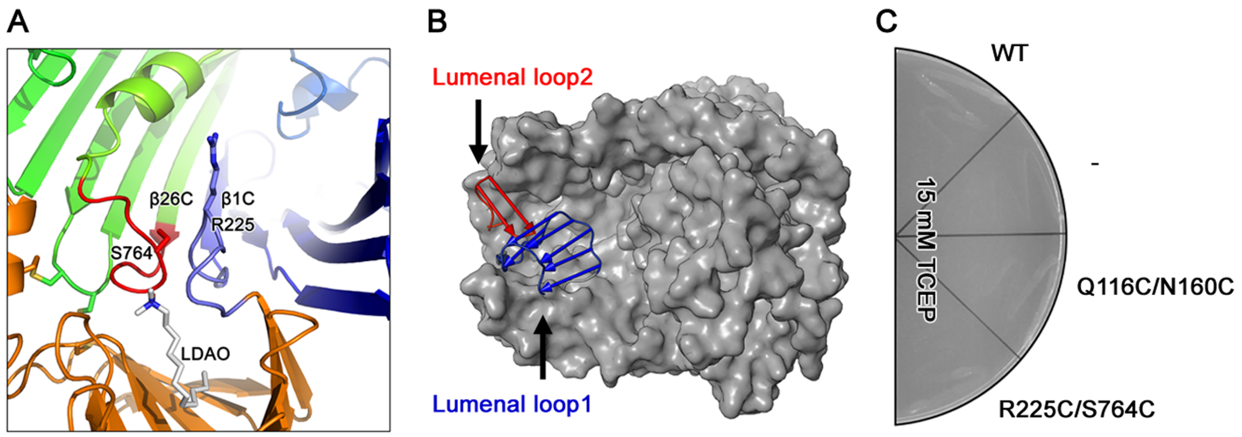

Figure S3, related to Figure 3. The lumen gate of LptD, and The *E. coli* cells with the LptD wild type and cysteine mutants Q116C/N160C and R225C/S764C are killed or inhibited by 15 mM TCEP. (*A*) The lumen gate are two loops in blue and red. There is a LDAO molecule, LPS's mimetics, in front of the lumen gate. Residues S764 and R225 are shown in stick. (*B*) The switch of the lumen gate. The arrows show the lumen gate from an open position to a closed position, which is based on the LptD/E structures of *S. typhimurium* LT2 and *S. flexneri*. (C) The TCEP at 15 mM may break the disulfide bond between C173 and C727, which is in the membrane and essential for the vitality of *E. coli*.

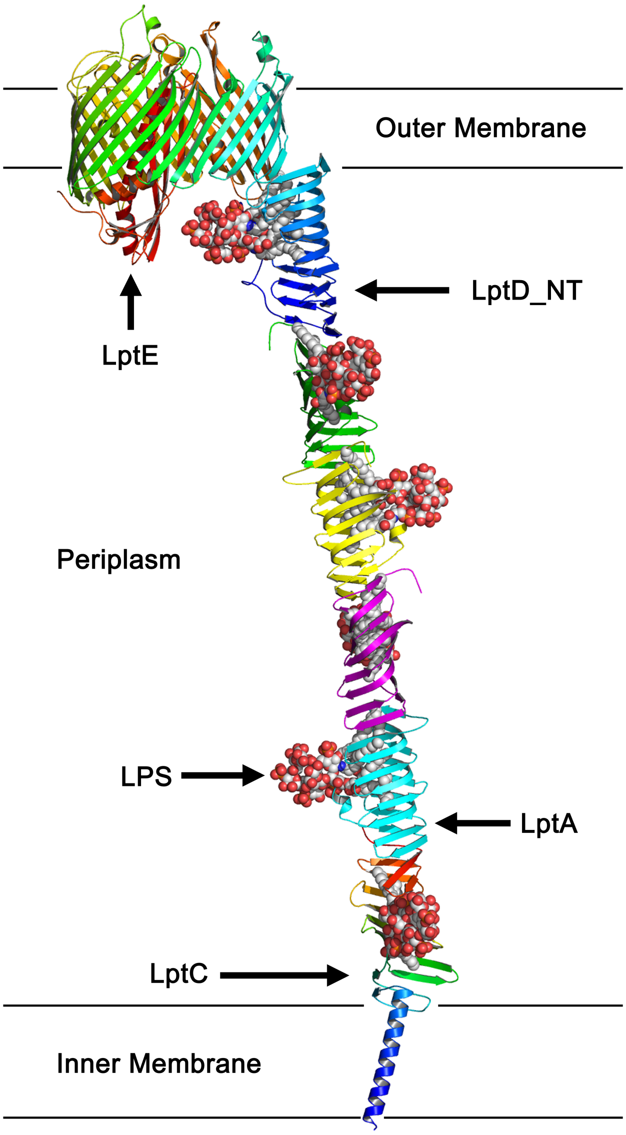

Figure S4, related to Figure 4. **The LPS transport slide formed by LptC, LptA and LptDE across the periplasm**. The slide contains one LptC, four LptA and one LptD molecule(s). The slide rotates ~360° from the LptC to LptD. The LPS molecules are modeled in the slide to show the rotation of the slide.

Video S1, related to Figure 2. The molecular dynamics simulation of oxidized LptD/E complex. The β1C in red and β26C in green. The hydrophobic residues in the intramembrane hole are in blue.
